# Cerebral rheumatoid vasculitis: an integrated analysis of case presentation, literature review, and prognostic stratification

**DOI:** 10.3389/fimmu.2025.1683920

**Published:** 2025-10-28

**Authors:** Yinghui Zhang, Xuefeng Zhai, Li Ning, Juan Yang, Liu Yang, Jian Li, Shuhong Chi, Boya Ma, Liu Lu, Zhao Li, Yang Xiao

**Affiliations:** ^1^ Department of Neurology, General Hospital of Ningxia Medical University, Yinchuan, China; ^2^ Department of Pathology, General Hospital of Ningxia Medical University, Yinchuan, China; ^3^ Department of Neurosurgery, General Hospital of Ningxia Medical University, Yinchuan, China; ^4^ Department of Radiology, General Hospital of Ningxia Medical University, Yinchuan, China; ^5^ Department of Rheumatology and Immunology, General Hospital of Ningxia Medical University, Yinchuan, China

**Keywords:** rheumatoid arthritis, cerebral vasculitis, biopsy, immunosuppression, mortality

## Abstract

This study integrated a comprehensive literature review spanning from 1951 to 2025 with a histologically confirmed case of cerebral rheumatoid vasculitis (CRV) in order to improve the management of this rare and severe complication of rheumatoid arthritis (RA). By analyzing 37 CRV cases, we observed a striking decline in mortality after the 1980s, with an odds ratio (OR) of 33.07. The combination of immunosuppressants and corticosteroids significantly enhanced treatment outcomes, showing 81.8% efficacy compared to a 63.6% mortality rate without immunosuppressants. Based on these findings, we developed the CRV diagnostic workflow to aid early detection. Our results emphasize the importance of vigilance for neurological symptoms in RA patients and highlight the need for future research to optimize treatment protocols and reduce long-term disability.

## 1 Introduction

Cerebral rheumatoid vasculitis (CRV), a rare central nervous system (CNS) complication of rheumatoid arthritis (RA), presents considerable diagnostic and therapeutic challenges due to its complex pathology, non-specific clinical features, and absence of reliable biomarkers. Rheumatoid vasculitis (RV), a non-infectious inflammatory condition, affects approximately 1% to 5% of RA patients (1, 2), typically involving small-to-medium vessels. When RV involves the CNS, it is classified as CRV. Although CRV accounts for only 1% to 8% of all RV cases (3, 4), its necrotizing vascular inflammation often leads to irreversible neurological injury or death, underscoring the need for early recognition and treatment (5). Diagnosis remains difficult because imaging findings are often non-specific, biopsy is invasive, and clinical presentations vary widely, including headache, cognitive impairment, and focal deficits, which frequently result in delayed or missed diagnosis. Most cases are identified only after permanent neurological damage has occurred. Treatment evidence remains limited; while corticosteroids combined with immunosuppressants such as cyclophosphamide have shown benefit in some case reports (6, 7), supporting data are largely derived from retrospective analyses or small series. No standardized therapeutic protocol currently exists, although biological agents have also demonstrated potential in isolated reports (2, 4, 8, 9). In summary, CRV is a severe RA complication lacking evidence-based diagnostic and treatment frameworks, forcing clinicians to rely on individualized judgment. This article presents a biopsy-confirmed, successfully managed CRV case and reviews relevant literature through 2025, with the aim of improving clinical recognition and proposing structured management strategies.

## 2 Case report

### 2.1 Part 1 (patient information)

A 64-year-old woman with a 30-year history of RA was admitted on September 10, 2023, due to 10 days of progressive drowsiness and fatigue, which worsened with left-sided limb weakness over 3 days and culminated in generalized tonic–clonic seizures 1 day before admission. Her RA had been managed with long-term corticosteroids, which were initially used regularly. She also had a 10-year history of hypertension, controlled with sacubitril–valsartan and amlodipine.

Nine days before admission, she developed hypersomnolence, apathy, reduced speech, and fatigue. Within 3 days, her condition progressed to include left limb weakness, impaired consciousness, aphasia, mild fever, and urinary incontinence. Cranial CT revealed bilateral temporo-occipital hypodensities. One day prior to admission, she experienced six seizures, each lasting 1–2 minutes, followed by coma and fever peaking at 38.7°C.

On admission, she exhibited low-grade fever (37.3°C) and hypertension (170/100 mmHg). RA-related deformities were observed in her hands and feet, accompanied by joint tenderness but no subcutaneous nodules. Neurologically, she was in a mild coma and unresponsive to noxious stimuli, with isocoric pupils and sluggish light reflexes. Muscle tone was normal, and there were no signs of meningeal irritation or pyramidal tract involvement.

### 2.2 Part 2 (laboratory tests)

Hematological analysis demonstrated a leukocyte count of 6.11 × 10^9^/L with neutrophilia (81.4%) and lymphopenia (11.5%), alongside mild reductions in erythrocyte indices, including red blood cell count (3.27 × 10^12^/L), hemoglobin (105 g/L), and hematocrit (32.5%); platelet levels remained normal at 191 × 10^9^/L. Inflammatory markers were significantly elevated: procalcitonin (0.190 ng/mL; normal ≤0.094 ng/mL), interleukin-6 (230 pg/mL; normal ≤7 pg/mL), and high-sensitivity C-reactive protein (44.3 mg/L; normal <10 mg/L). Tumor markers carcinoembryonic antigen (CEA), alpha-fetoprotein (AFP), CA125, CA153, CA199, and neuron-specific enolase (NSE) were all negative; infectious serology showed no evidence of syphilis or HIV with sterile blood cultures. Immunological rheumatological parameters are detailed in **Table 1**. Cerebrospinal fluid (CSF) analysis revealed colorless/transparent fluid under normal opening pressure (90 mmH_2_O), with elevated IgG (533 mg/L) and protein (2.20 g/L) levels, while glucose and chloride were normal. A lymphocytic-predominant pleocytosis was noted (135 cells/mm^3^; lymphocytes 60%, monocytes 24%, neutrophils 13%, and plasma cells 3%), and both microbiological cultures and cytopathology (Alcian blue staining) yielded negative results.

**Table 1:** Rheumatoid factor test results.

Positive (increased)	Negative (decreased)
Rheumatoid factor (RF) 563 IU/mL (normal: 0–15.9 IU/mL)	Complement C3 0.81 g/L (normal: 0.9–1.8 g/L)
Anti-cyclic citrullinated peptide (anti-CCP) antibody >200 RU/mL (normal: <5 RU/mL)	Sjögren's Syndrome A (SSA)
Erythrocyte sedimentation rate (ESR) 56 mm/h (normal: 2–38 mm/h)	Sjögren's Syndrome B (SSB)
Anti-keratin antibody (AKA)	Smith (Sm)
	U1 Ribonucleoprotein (U1RNP)
	Antinuclear antibodies (ANAs)
	Anti-neutrophil cytoplasmic antibody (ANCA) profile
	Anti-double-stranded DNA antibody (anti-dsDNA)
	Lupus anticoagulant (LA)

**Figure 1:**
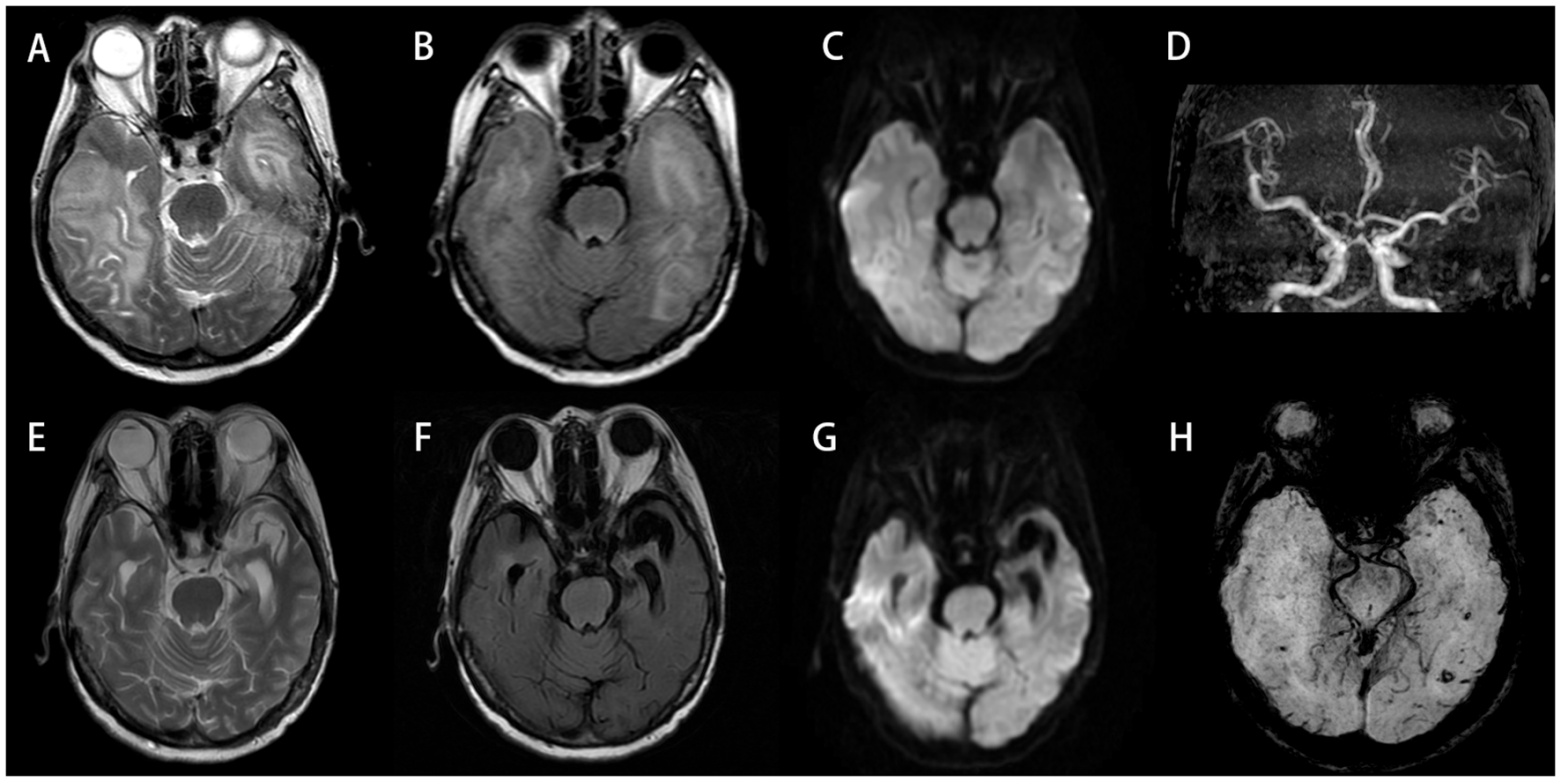
**(A–D, H)** Initial scan (September 13, 2023). Bilateral temporal and occipital lobes demonstrate extensive abnormal signals: hyperintensity on T2-Weighted Imaging (T2WI) **(A)**, with mild hyperintensity on T2/FLAIR **(B)**, but no restricted diffusion on DWI **(C)**. MRA reveals intracranial atherosclerotic changes, featuring segmental stenosis in the right middle cerebral artery M1 segment **(D)**. SWI shows scattered punctate hypointensities in bilateral temporo-occipital regions, suggestive of microhemorrhages **(H)**. **(E–G)** Follow-up scan (July 17, 2024; 10-month post-baseline): previous abnormal signals in bilateral temporo-occipital regions have largely resolved, with encephalomalacia formation in the left temporal lobe. DWI hyperintense areas exhibit interval expansion compared to September 2023 baseline. Significant progression of cerebral atrophy is noted. DWI, diffusion-weighted imaging; MRA, magnetic resonance angiography; SWI, susceptibility-weighted imaging.

**Figure 2:**
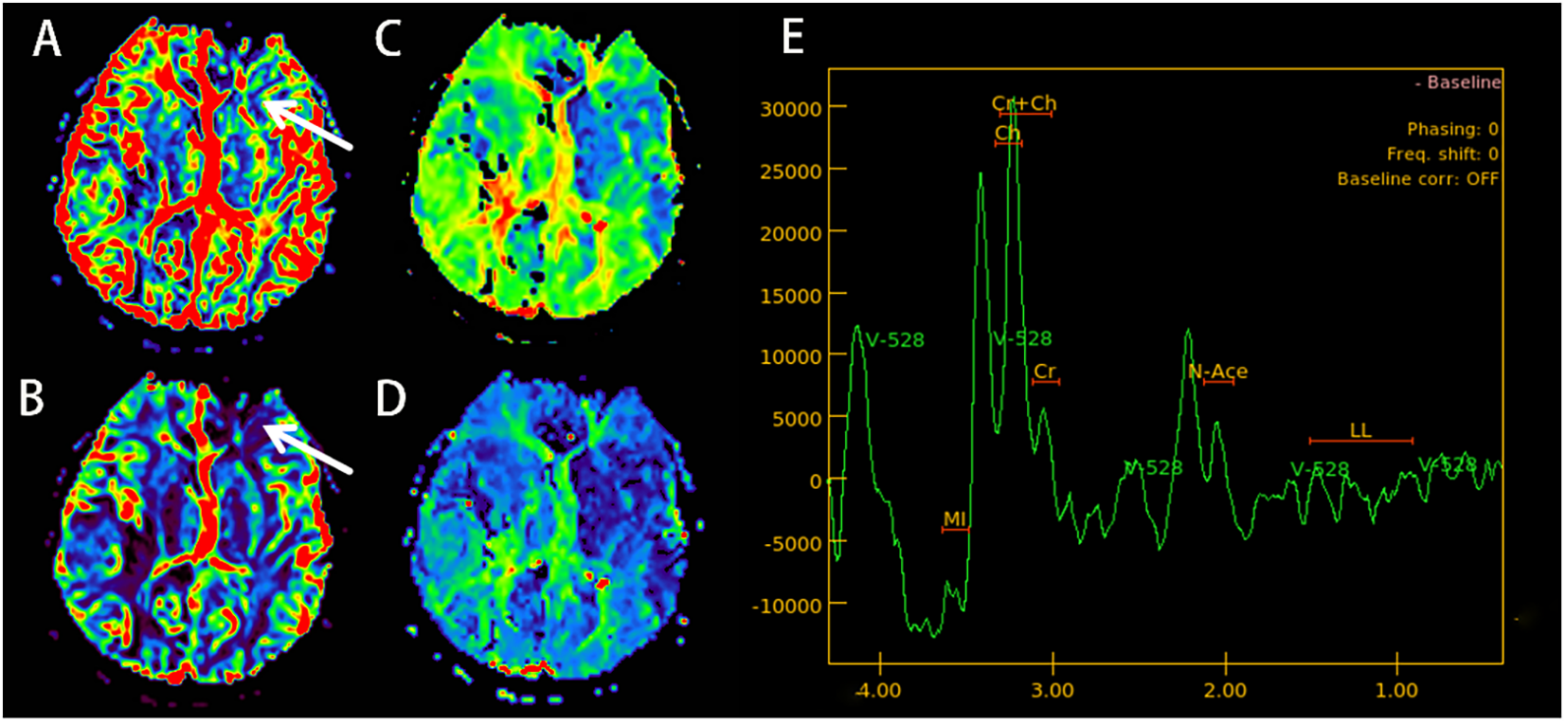
(September 13, 2023; PWI+MRS): cerebral blood volume (CBV) imaging shows a decrease in the blood volume in the left frontal lobe (arrow) **(A)**. Cerebral blood flow (CBF) imaging shows a decrease in cerebral blood flow in the left frontal lobe (arrow) **(B)**. Time to peak (TTP) **(C)** and time to maximum (Tmax) **(D)**, indicating that blood flow perfusion time in the right frontal and temporal lobes is longer than on the left side. MRS **(E)** shows a slight increase in the Choline (CHO) peak in the right temporal lobe area of interest, while the NAA peak drops. PWI, perfusion-weighted imaging; MRS, magnetic resonance spectroscopy; NAA, *N*-acetylaspartate.

Electroencephalogram (EEG) demonstrated moderate diffuse abnormalities characterized by theta rhythm in the background activity and frontal-predominant sharp/slow waves. Magnetic resonance imaging (MRI) revealed extensive coalescing lesions in the bilateral parietal, temporal, and occipital lobes extending to the basal ganglia, exhibiting T1 hypointensity, T2 hyperintensity, and mild T2/FLAIR hyperintensity, but no hyperintensity on diffusion-weighted imaging (DWI) (**Figures 1A–C**). Vascular studies identified significant intracranial atherosclerosis, with magnetic resonance angiography (MRA) showing right middle cerebral artery M1-segment stenosis (**Figure 1D**), while magnetic resonance venography (MRV) confirmed left transverse/sigmoid sinus narrowing. Susceptibility-weighted imaging (SWI) detected scattered microhemorrhagic foci in temporal/occipital regions (**Figure 1H**). Perfusion-weighted imaging (PWI) demonstrated hemodynamic disturbances characterized by reduced cerebral blood volume (CBV) and cerebral blood flow (CBF) in the left frontal lobe, as indicated by an arrow, along with prolonged perfusion times in the right frontotemporal regions, evidenced by time to peak (TTP) and time to maximum (Tmax) parameters compared to the left side (**Figures 2A–D**). Magnetic resonance spectroscopy (MRS) of the right temporal lobe exhibited metabolic derangement with elevated choline and reduced *N*-acetylaspartate (NAA) peaks (**Figure 2E**).

Further diagnostic evaluations definitively excluded alternative etiologies, including a negative CSF PCR for JC virus, the absence of pathogenic sequences via metagenomic capture (MateCAP) testing, and non-reactive serum/CSF antibodies targeting AQP4, MOG, and autoimmune encephalitis-associated antigens.

### 2.3 Part 3 (treatment, outcome, and follow-up)

**Figure 3:**
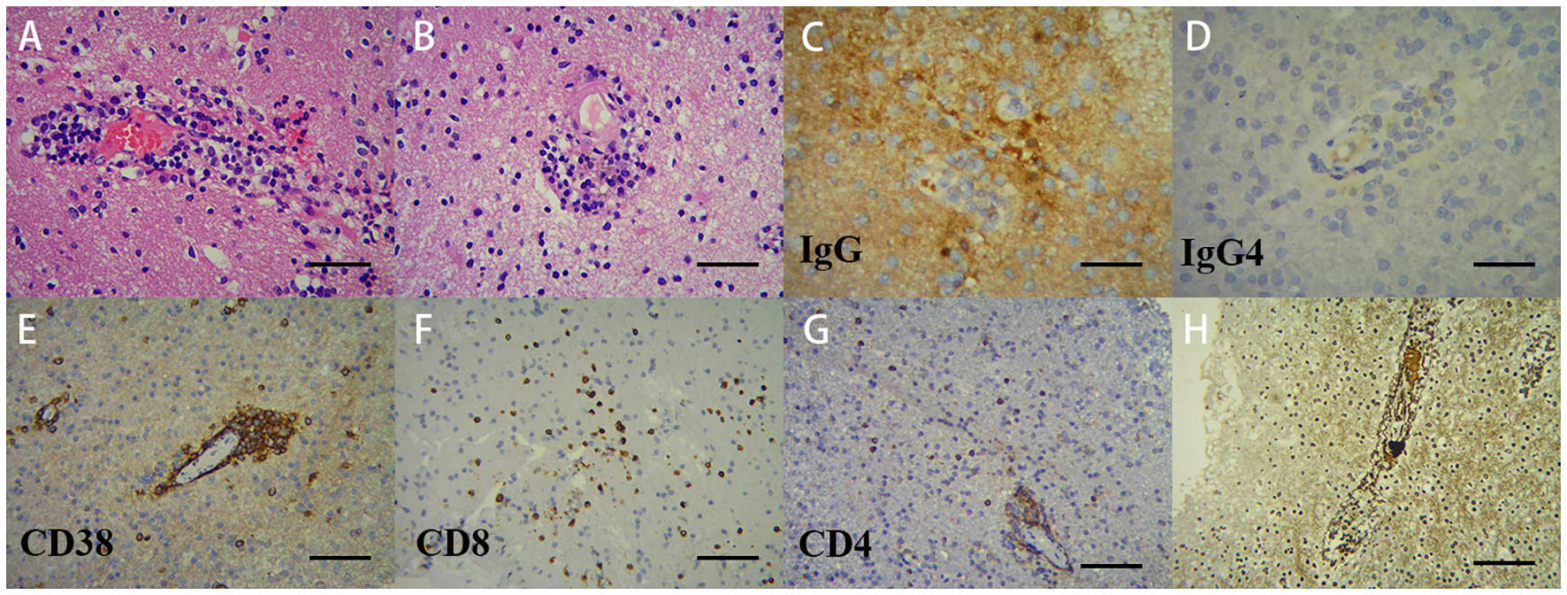
Pathological analysis of the right temporal lobe lesion demonstrates vasculitic inflammation. Microscopic examination: (**A**; ×200) lymphoplasmacytic infiltration with microthrombi in small-to-medium vessel walls; (**B**; ×200) hyaline vascular degeneration with transmural inflammation (lymphocytes, plasma cells, and neutrophils), adjacent microinfarcts, and activated microglial proliferation; (**C, D**; ×400) IgG+ and IgG4−. Immunohistochemistry: (**E**; ×200) CD38^+^ plasma cells predominate within vascular lesions; (**F, G**; ×200) CD8^+^/CD4^+^ T cells predominantly perivascular. Reticulin stain **(H)** confirms architectural destruction, supporting immune-mediated vasculitis.

Twelve days post-admission, a right temporal lobe brain biopsy was performed under general anesthesia. Histopathological examination revealed brain parenchyma gliosis with focal softening, as well as vascular wall necrosis accompanied by perivascular lymphocytic, plasmacytic, and neutrophilic infiltration. Immunohistochemical staining was positive for IgG but negative for IgG4 (**Figure 3**). CNS vasculitis was confirmed. Given her RA history, CRV was diagnosed.

Treatment composition: The patient’s treatment regimen consisted of initial pulse therapy with intravenous methylprednisolone sodium succinate (500 mg/day for 3 days), followed by a stepped reduction to 240 mg/day for 3 days and then 120 mg/day for 3 days. Therapy was then transitioned to oral prednisone acetate at 60 mg/day, in combination with oral cyclophosphamide tablets (100 mg every other day). For secondary epilepsy, the patient was additionally treated with levetiracetam tablets at a dose of 0.5g twice daily.

Outcome: Gradual fever reduction and consciousness improvement began immediately. Full consciousness was restored on Day 3; language/limb function recovered by Day 14, at which time the patient was discharged home. No further epileptic seizures were observed throughout the treatment course. Discharge medication consisted of oral prednisone 40 mg/day (tapered slowly), cyclophosphamide 100 mg every other day, and levetiracetam 0.5g twice daily for seizure prophylaxis.

Significant neurological improvement was observed during hospitalization. The marked transition from obtundation to alert consciousness, accompanied by complete motor recovery and functional independence restoration, is empirically documented in **Table 2**.

**Table 2:** Evolution of neurological status: admission-to-discharge comparison.

Parameters	Admission (Sep 10, 2023)	Discharge (Oct 7, 2023)
Consciousness	Obtundation (GCS 3)	Alert (Glasgow Coma Scale (GCS) 15)
Motor response	Absent	Normal (5/5 all limbs)
Functional status	mRS 5	mRS 0

One month after discharge, she returned for a follow-up appointment, reporting that she felt mentally well and had no issues. A follow-up MRI+DWI conducted on November 2, 2023, showed that the lesions in the temporal, parietal, and occipital lobes had decreased slightly in size compared to those in the previous scan on September 13, 2023. Ten months after discharge, another follow-up MRI+DWI indicated that the original abnormal signals in the bilateral temporal and occipital lobes had largely resolved; however, significant brain atrophy was observed (**Figures 1E–G**). At that time, the dosage of prednisone acetate had been reduced to 10 mg once daily, and cyclophosphamide was administered at 50 mg every other day. Aside from polyarticular pain and poor sleep, the patient did not report any other major issues.

## 3 Method

### 3.1 Study design and case identification (this study employed a dual-method approach)

#### 3.1.1 Case report

Prospective documentation was conducted of a histopathologically confirmed CRV case treated at the General Hospital of Ningxia Medical University (September 2023–July 2024).

#### 3.1.2 Literature review

A comprehensive analysis was conducted of all published CRV cases from 1951 to 2025 to establish a pooled cohort.

### 3.2 Data collection

#### 3.2.1 Literature cohort

PubMed, EMBASE, CNKI, and Web of Science were systematically searched (January 1951–June 2025) using the following keywords: “cerebral rheumatoid vasculitis”, “RA central nervous system complication”, “rheumatoid vasculitis brain”, and “necrotizing vasculitis RA”.

#### 3.2.2 Inclusion criteria

American College of Rheumatology/ European Alliance of Associations for Rheumatology (ACR/EULAR) diagnostic criteria for RA were met; CRV was confirmed by pathology (brain biopsy) or imaging/clinical combination; demographic, diagnostic, and prognostic data were relatively complete.

#### 3.2.3 Exclusion criteria

Patients with systemic lupus erythematosus (SLE), Sjogren’s syndrome (SS), and other autoimmune diseases were removed, as well as infectious, tumor, or drug secondary vasculitis cases.

A total of 36 historical cases met the criteria and were combined with the index case (n = 37).

### 3.3 Data extraction and variables analyzed

Demographics included age, sex, RA duration, and serological markers [rheumatoid factor (RF), C-reactive protein (CRP), and erythrocyte sedimentation rate (ESR)].

Clinical features included neurological symptoms (seizures, coma, focal deficits, etc.).

Diagnostic workup included neuroimaging (MRI/MRA), CSF analysis, and histopathology.

Treatment included regimens (corticosteroids ± immunosuppressants/biologicals), dosing, and duration.

Outcomes included survival and death.

### 3.4 Statistical analysis (data were analyzed using SPSS 29.0)

#### 3.4.1 Descriptive statistics

Continuous variables were presented as mean ± SD or median Interquartile Range (IQR), and categorical variables as frequencies (%).

#### 3.4.2 Comparative analyses

Mortality differences were determined using Fisher’s exact test. Temporal mortality trends (pre- vs. post-1980s) were determined using the Cochran–Armitage test and Fisher’s exact test. Treatment efficacy (monotherapy vs. combination) was determined using chi-square tests.

### 3.5 Ethical considerations

The index case provided written informed consent. The study protocol was approved by the Ethics Committee of Ningxia Medical University (Approval No. NMU-KYLL-2025-1707). Literature data were anonymized and publicly available; no additional ethics approval was required.

## 4 Discussion and literature review

CNS inflammation is an uncommon RA extra-articular manifestation, primarily comprising non-specific meningeal inflammation, rheumatoid nodules, and vasculitic changes (1, 3, 10, 11). CRV represents a rare but life-threatening form. Before the 1990s, CRV was poorly understood, with most diagnoses made post-mortem (8). Advances in neuroimaging and serology have improved the premortem diagnosis of CRV; however, cases remain rare, and no treatment consensus exists. This review summarizes current literature on CRV, with emphasis on its epidemiology, etiology, diagnostic approaches, management strategies, and prognostic outcomes.

### 4.1 Epidemiology and clinical features

In their 1951 description of the first documented CRV case, Pirani and colleagues (12) reported a 22-year-old male patient with RA onset at age 6. The patient ultimately developed delirium and seizures, leading to death; autopsy revealed basilar artery Polyarteritis nodosa. This case led to the initial identification and naming of CRV. It is recognized as a rare and severe disease that poses diagnostic challenges. According to the author’s review of past literature, a total of 37 cases have been reported: 36 cases were previously documented, and one case is presented in this report (original data are presented in **Supplementary Table 1** and **Table 2**). Consequently, the epidemiological data remain unclear.

**Figure 4:**
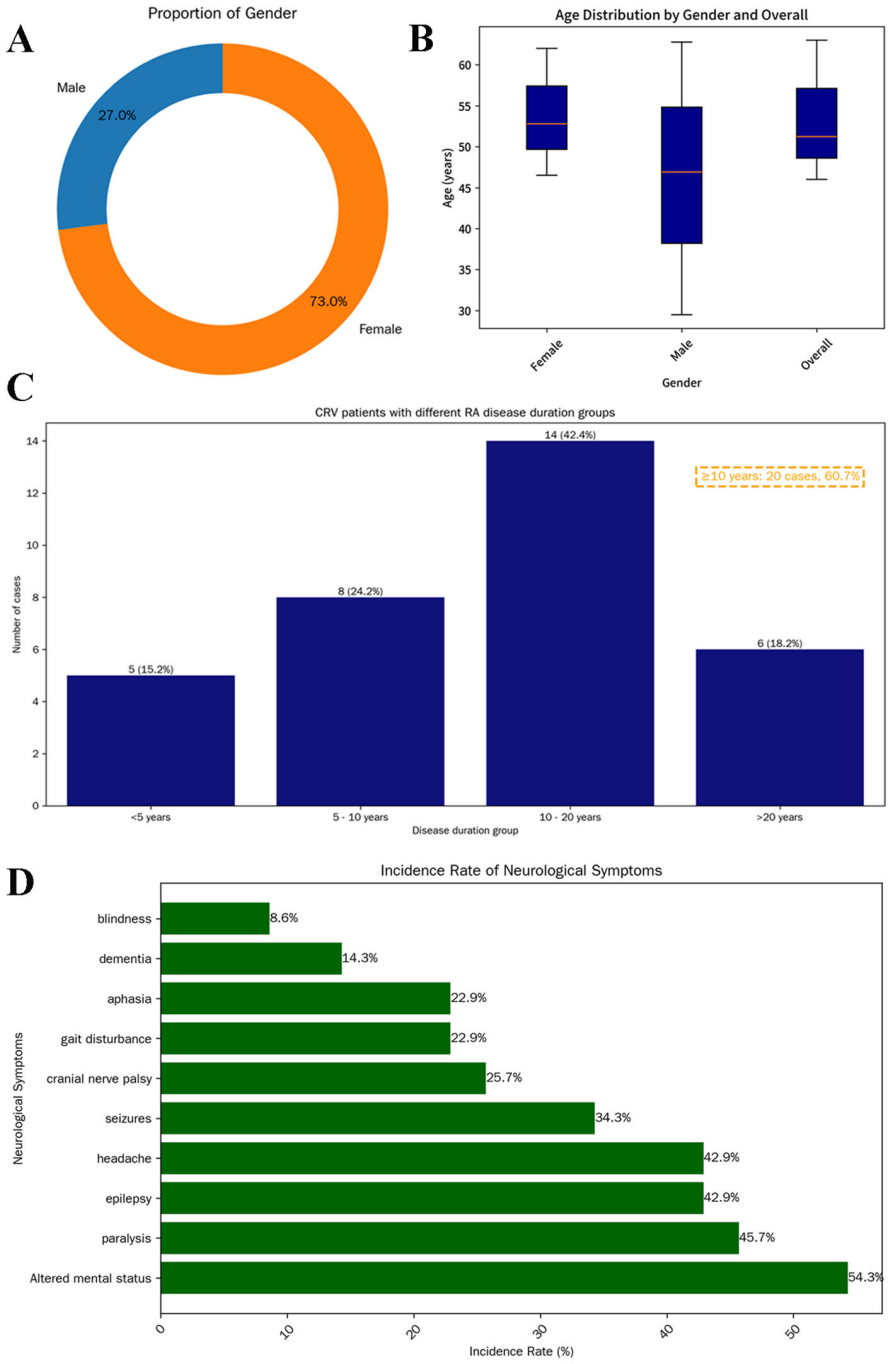
Demographic characteristics of 37 patients with CRV. Donut chart showing sex distribution: male (blue, 10, 27%) and female (orange, 27, 73%) **(A)**. Box plot illustrating age distribution by sex. The median age of all patients was 51 years (IQR: 46–63); females: 53 years (IQR: 46.5–62); males: 47 years (IQR: 29.5–62.8) **(B)**. Distribution of RA history duration among CRV patients (n = 33). A total of 20 patients (60.7%) had a disease duration exceeding 10 years **(C)**. Prevalence of various neurological symptoms in patients **(D)**. CRV, cerebral rheumatoid vasculitis; RA, rheumatoid arthritis.

Based on a literature review of 37 cases of CRV, we found that the disease exhibits a significant gender predilection, which is female predominance (73.0%, 27/37; median age 51 years, range 16–81) (**Figures 4A, B**). This 3:1 female-to-male ratio mirrors general RA epidemiology (13), suggesting that estrogen or immune mechanisms influence CRV development.

Research confirms that RV typically manifests in severe, seropositive RA with poorly controlled disease (1, 4, 6, 14). RV correlates with erosive joint damage and occurs after prolonged RA duration [n = 33, median 12 years, 60.7% (20/33) ≥10 years], supporting chronic inflammation as a key risk factor (**Figure 4C**). This aligns with the hypothesis that CRV represents a late complication of uncontrolled RA, likely from cumulative immune complex-mediated endothelial injury (15). The presented case, with a 30-year RA duration and markedly elevated RF (563 vs. mean 302.2 ± 256.2 IU/mL in CRV), exemplifies this association and reinforces the link between RF levels and RV severity (literature reports 76.2% RF positivity in CRV).

RV frequently occurs in patients with RA after prolonged corticosteroid use, as established by Dalila Mrabet et al. (3). The historical development includes Kemper’s (15) landmark 1957 study of 52 RV patients, which proposed that corticosteroids accelerated necrotizing vasculitis onset (16). This led to a decade-long belief in their causative role. However, pivotal case reports by Watson (1977) (17), Ramos (1975) (18), and Singleton (1995) (19) documented RV in corticosteroid-naïve patients, challenging the paradigm that all RV cases require prior steroid exposure. Among 37 CRV patients analyzed, 19 (51.4%) had prior glucocorticoid exposure. Medication patterns among these patients included treatment discontinuation before disease onset (one case, 5.3%), regular use (nine cases, 47.4%), and intermittent administration (seven cases, 36.8%), while treatment details remained unknown in two cases (10.5%). These heterogeneous patterns preclude definitive conclusions regarding glucocorticoid therapy as a potential risk factor for CRV development, warranting further investigation.

The neurological manifestations of CRV demonstrate marked heterogeneity depending on affected vascular territories. Headache and altered mental status represent the most frequently reported symptoms in established literature (3). Additional features may include seizures, cranial neuropathies, visual loss, motor deficits (e.g., paralysis and gait disturbances), aphasia, and cognitive impairment resembling encephalopathy (20–22). Clinical evolution may occur with disease progression (3, 23). In the current cohort (**Figure 4D**), neurological involvement was documented in 35 of 37 patients (94.6%), with mental status changes (54.3%), paralysis (45.7%), epilepsy (42.9%) and headache (42.9%) being predominant, potentially reflecting a predilection for cerebral cortical involvement.

### 4.2 Diagnostic challenges and integrated approach

Laboratory assessment of CRV focuses on systemic inflammation and immune dysregulation markers, although diagnostic interpretation requires caution. Elevated RF, increased ESR, and decreased complement C3 contribute to diagnosis, whereas anti-cyclic citrullinated peptide (anti-CCP) antibody demonstrates no established clinical utility (24, 25). This cohort analysis (n = 37) revealed RF positivity in 76.2% (16/21) (mean 302.2 ± 256.2 IU/mL) and universal ESR elevation (mean 75.04 ± 33.16 mm/h), indicating acute systemic inflammation potentially exacerbating vascular endothelial injury. Reduced C3 in 25% (3/12) of patients supports immune complex-mediated complement consumption, consistent with histopathological evidence of vascular IgG/C3 deposition. However, in rheumatoid meningitis (RM)—a condition distinct from but related to RA, as reported by Nissen et al. (26) and Higashida-Konishi et al. (27)—the Anti-Citrullinated Protein Antibody (ACPA) index has been proposed as a potential diagnostic biomarker and may indicate CNS involvement in RA. In the present case, the patient exhibited markedly elevated anti-CCP antibody levels. By contrast, there are limited data regarding anti-CCP antibodies in previously published CRV cases. Thus, further investigation is warranted to determine its utility as a diagnostic biomarker for CRV. If the final results show no significant positive findings, it may suggest that the pathogenesis of this disease is independent of classical RA joint destruction pathways. Additional studies suggest that 29% of CRV patients exhibit antinuclear antibody (ANA) positivity, possibly reflecting a serological risk profile (28, 29). However, low historical detection rates for these inflammatory markers preclude definitive assessment of their individual or combined diagnostic utility. Future multicenter cohorts with standardized biomarker profiling and expanded sample sizes are warranted to address this gap.

CSF analysis in CRV may show lymphocytic pleocytosis and elevated protein levels, as observed in some cases; however, these findings are non-specific. Despite their limited disease specificity, CSF studies remain valuable in the diagnostic workup to exclude alternative infectious, inflammatory, or malignant disorders.

MRI serves as a crucial tool for evaluating neurological symptoms in RA patients. Based on previous literature, CRV typically presents with multifocal intracranial white matter lesions affecting multiple lobes, most commonly the frontal, temporal, and occipital regions. Infarctions and hemorrhagic foci may coexist (1, 28). MRA reveals cerebral arterial narrowing and irregular contours indicative of stenosis/occlusion, which may lead to misdiagnosis as non-inflammatory vascular events (the imaging findings of 37 patients are summarized in **Supplementary Table 3**). Notably, our representative case showed confluent bilateral temporo-occipital lesions involving both white and gray matter. This cross-territorial distribution pattern helped exclude cerebral infarction during initial differential diagnosis (**Figures 1A, B**). MRA demonstrated stenosis in the M1 segment of the right middle cerebral artery (**Figure 1D**). CRV may mimic infectious or neoplastic processes but typically shows minimal/no enhancement and lacks significant mass effect despite extensive involvement, serving as key differentiating features.

Limited prior reports—notably the only documented PWI/MRS study in CRV—demonstrated reduced CBF without CBV elevation in lesions (24). MRS indicated unaltered choline but diminished NAA peaks (24). Our case corroborates these findings: perfusion imaging revealed decreased CBF with prolonged TTP, alongside paradoxically elevated CBV in the left frontal regions, suggesting blood–brain barrier disruption. MRS of the right temporal lesion showed mildly increased choline and decreased NAA (**Figure 2**). This convergence with 2022 reports suggests a characteristic CRV microcirculatory impairment pattern—primarily manifesting as multifocal hypoperfusion in terminal arteriole territories, evidenced by reduced CBF relative to contralateral regions. CBV exhibits dynamic evolution: initial collateral recruitment maintains near-normal values, progressive ischemia prompts CBV decline, and blood–brain barrier disruption induces paradoxical local elevation. Neuronal loss consistently accompanies these changes. Larger cohort validation remains essential.

Before the 1990s, CRV complications were underrecognized due to a lack of biomarkers and imaging features (30). Histopathological diagnosis (achieved in 48.6% of reported cases) reveals necrotizing vasculitis of small-to-medium vessels characterized by perivascular inflammatory cuffing, endothelial swelling, nuclear dust, and fibrinoid necrosis (33.3%). Vascular fibrosis or amyloidosis may co-occur (22.2%). These changes cause vascular occlusion, thrombosis, and subsequent ischemic lesions (58.3% on imaging) or beaded stenosis (28.6%). Given biopsy invasiveness and low patient acceptance, clinical diagnosis relies on RA history, neurological symptoms (e.g., seizures and altered consciousness), and exclusion of alternative etiologies (28).

Initial diagnostic considerations in this immunosuppressed patient presenting with fever, seizures, and encephalopathy centered on progressive multifocal leukoencephalopathy (PML), given the extensive non-enhancing white matter lesions lacking diffusion restriction (8, 31). PML was definitively excluded by two separate negative CSF JC virus PCR assays. Comprehensive evaluation, including CSF profiling, negative autoimmune encephalopathy antibody panels, and negative metagenomic next-generation sequencing, further eliminated other inflammatory or infectious etiologies. The low probability of neoplastic processes was supported by three key factors: absence of systemic malignancy, symmetric non-expansile lesions inconsistent with metastases, and lesion features atypical for CNS lymphoma or astrocytoma. Specifically, CNS lymphoma usually presents with periventricular involvement, whereas astrocytoma typically exhibits poorly defined margins and mass effect.

In addition, in the diagnostic evaluation of RA patients with CNS symptoms, RM represents a critical entity to distinguish from CRV (32–34). Although both conditions can present with overlapping clinical features, their underlying pathophysiology and radiological signatures differ (22, 34). CRV primarily manifests as parenchymal injury and the characteristic “string-of-beads” stenosis on MRA (22). In contrast, RM involves inflammation and rheumatoid nodule deposition within the meninges (10). Accordingly, RM typically exhibits leptomeningeal or pachymeningeal enhancement on post-contrast T1-weighted MRI, often predominant in the frontoparietal regions (35). A recently described imaging clue for RM is the “mismatch DWI/FLAIR” sign, which refers to patchy diffusion restriction without corresponding FLAIR hyperintensity (10, 33). Notably, this mismatch pattern was reversed in our case: the cerebral lesions showed significant T2/FLAIR hyperintensity but no hyperintensity on DWI. This fundamental difference in imaging underscores the radiologic distinction between the two entities. Therapeutically, while both conditions require immunosuppression, RM often shows a favorable response to high-dose corticosteroids and conventional Disease-Modifying Antirheumatic Drugs (DMARDs) (36, 37), whereas CRV may necessitate more aggressive regimens, such as cyclophosphamide. Therefore, incorporating RM into the differential diagnosis is essential for guiding appropriate imaging interpretation and treatment selection.

Based on the patient’s clinical, laboratory, and imaging findings, infectious and neoplastic etiologies were systematically excluded. A diagnostic brain biopsy of the right temporal lobe was performed, which revealed features consistent with vasculitic inflammation. Histopathological examination showed lymphoplasmacytic infiltration with microthrombi in small-to-medium vessel walls, hyaline vascular degeneration, transmural inflammation, and associated microinfarcts with activated microglial proliferation. Immunohistochemistry was positive for IgG but negative for IgG4, effectively ruling out IgG4-related disease (**Figure 3**). These findings support a diagnosis of central nervous system vasculitis. In the context of the patient’s longstanding RA, the pathology was attributed to CRV.

The patient’s 30-year RA history, combined with multifocal non-enhancing lesions without significant edema, possible vascular beading, and CSF inflammation, raised suspicion for rheumatoid-associated CNS vasculitis—an inflammatory vasculopathy causing headaches, focal deficits, and cognitive impairment. Histopathological confirmation established the CRV diagnosis.

### 4.3 Treatment evolution and efficacy

Treatment consensus for this rare complication remains elusive. Before the 1990s, corticosteroid monotherapy showed limited efficacy (40% response rate among 10 patients). Rodriguez et al. (38) documented two CRV cases demonstrating marked clinical improvement following combined methylprednisolone and cyclophosphamide therapy, applying the protocol established by Cupps et al. (39) for glucocorticoid-refractory primary angiitis of the CNS. These pivotal reports fundamentally reshaped the therapeutic paradigm for CRV management. Analysis of 37 patients (**Table 3**) demonstrates that 81.8% (18/22) achieved clinical improvement with immunosuppressants, including cyclophosphamide (54.5%), methotrexate (22.7%), azathioprine (18.1%), rituximab (9.1%), or intravenous immunoglobulin (9.1%) combined with corticosteroids. Conversely, 63.6% (7/11) of fatal cases received no immunosuppressive therapy. The most effective regimen involves methylprednisolone pulses with cyclophosphamide, addressing both cerebrovascular and systemic RA manifestations. Furthermore, a recent study by Puéchal X et al. (40) demonstrated that nearly three-quarters of patients achieved complete remission of systemic rheumatoid vasculitis following rituximab treatment in routine clinical practice. This regimen offers the advantage of reducing the prednisone dosage by 40%–60% while helping prevent further neurological damage. However, due to heterogeneity among patients, larger-scale clinical trials are still needed to further evaluate its efficacy and safety.

**Table 3:** Clinical data on therapeutic approaches and clinical outcomes in patients with CRV (n = 37).

Modalities of treatment	Deaths	Improvement cases	Total
Unknown	2	0	2
Hormone therapy alone	7	6	13
Hormone + immunosuppressant	4	18	22
Total	13	24	37

CRV, cerebral rheumatoid vasculitis.

Notably, 50% (9/18) of cases were histologically confirmed as necrotizing vasculitis; however, the mortality rate in this subtype was consistent with that of non-necrotizing cases—both occurring in six of nine patients (66.7%). This suggests that, within this small sample, necrotic changes in vasculitis may not represent an independent risk factor for mortality. Larger-scale studies are warranted to validate the relationship between vasculitis subtypes and clinical outcomes.

**Figure 5:**
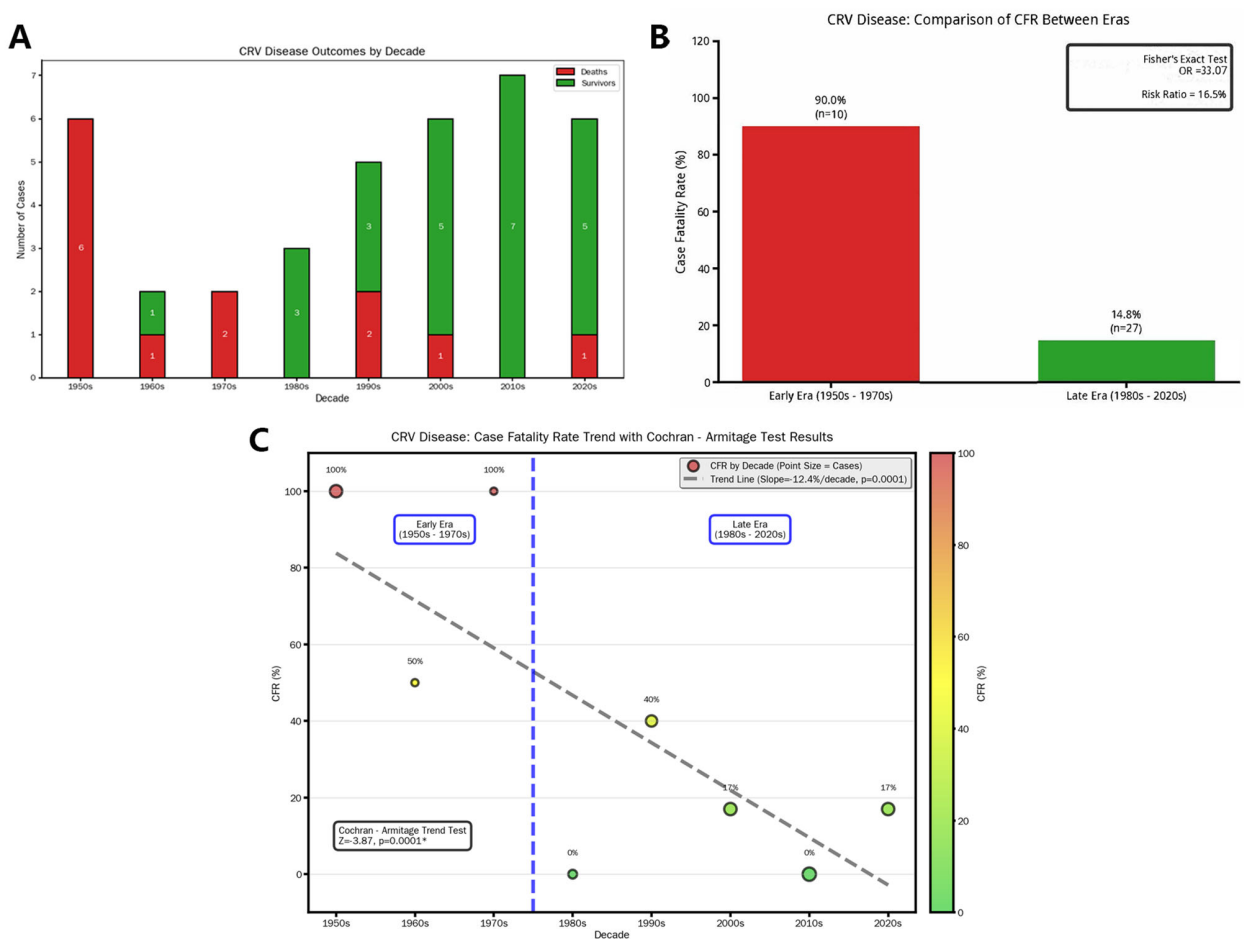
Outcomes of CRV treatment. **(A)** Bar chart showing the survival status of current patients across different decades, which shows a gradual decline in the death rate of CRV patients over time. Through epochal stratification at the 1980 demarcation, **(B)** Fisher’s exact test was employed to compare case fatality rates, revealing statistically significant inter-period disparity (p < 0.05). Quantitatively, mortality odds were substantially amplified in the pre-1980 cohort (Fisher’s exact test produced an OR of 33.07), whereas post-1980 fatalities represented only 16.5% of this historical benchmark—equivalent to an 83.5% relative risk reduction. **(C)** A significant decrease in the death rate for CRV patients over time using the Cochran–Armitage trend test (p < 0.05). CRV, cerebral rheumatoid vasculitis.

Cohort analysis (n = 37) demonstrated a significant decline in mortality (p < 0.05). The odds ratio (OR) was calculated as follows: among 10 patients treated before the 1980s, nine died and one survived; among 27 patients treated after the 1980s, four died and 23 survived (the list is shown in **Table 4**). The crude OR was 51.75 (95% CI: 5.07–528.10). To address the small sample size, we applied Fisher’s exact test with continuity correction, yielding an adjusted OR of 33.07, which still reflects a substantially higher odds of mortality in the pre-1980 era. By the post-1980 period, mortality risk decreased to only 16.5% of the historical burden, reflecting an absolute risk reduction of 83.5% between the two epochs (**Figure 5**). This prognostic improvement aligns with broader adoption of brain biopsies and corticosteroid-immunosuppressant combinations.

**Table 4:** Contingency table stratified by year 1980.

Group	Deaths	Survivals	Total
Pre-1980	9	1	10
Post-1980	4	23	27
Total	13	24	37

Exploratory Receiver Operating Characteristic (ROC) analysis suggested that neither RA history nor ESR alone demonstrated significant prognostic value in this cohort (AUCs near 0.5) (**Supplementary Figure 1**), underscoring the need for future larger studies with multivariate modeling to identify reliable predictors.

Our representative patient responded well to methylprednisolone (500 mg/day) with subsequent cyclophosphamide augmentation after initial refractory inflammation. Given the patient’s 30-year history of RA, the treatment plan was established in consultation with the Department of Rheumatology and Immunology. Following symptomatic improvement and discharge, the patient is receiving ongoing follow-up and management in the rheumatology clinic. In addition to the initial neurological treatment, the patient is regularly receiving tocilizumab injections. At 22-month follow-up, sustained clinical improvement, normalized inflammatory markers, and radiographic resolution were documented, illustrating regimen efficacy.

### 4.4 Diagnostic workflow and limitations

This comprehensive review confirms that CRV lacks specific diagnostic biomarkers or characteristic imaging findings. The disease manifests with atypical, rapidly progressive neurological symptoms, where longer symptom duration correlates with worse outcomes. Although current immunotherapies provide symptomatic relief, permanent neurological deficits may persist, and relapse is common without sustained treatment. Consequently, early histological confirmation via biopsy followed by aggressive immunosuppression becomes critical for prognosis optimization.

**Figure 6:**
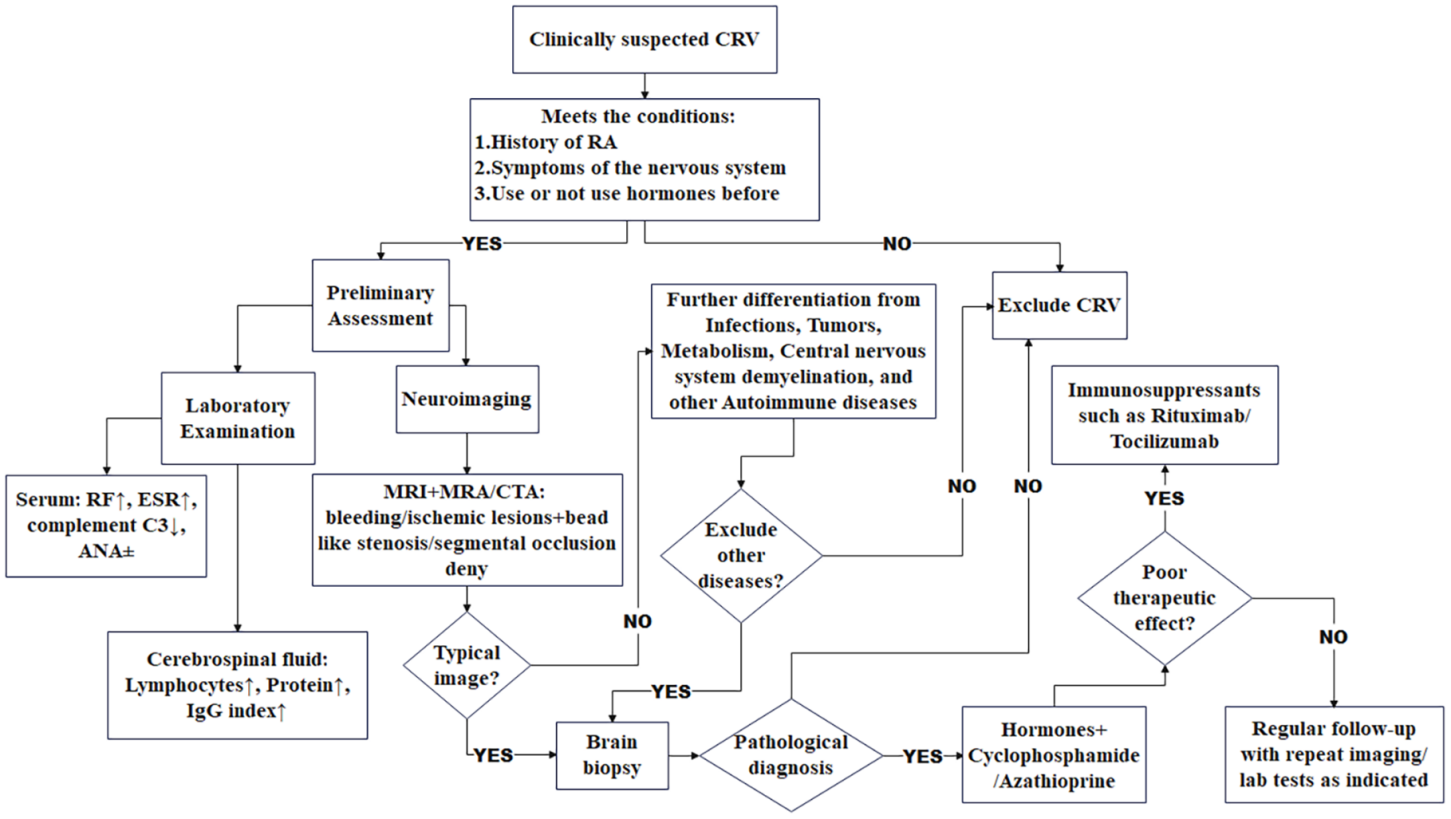
CRV diagnosis flowchart. CRV, cerebral rheumatoid vasculitis.

We have established a provisional diagnostic algorithm (**Figure 6**) to standardize evaluation. The proposed diagnostic algorithm for clinically suspected rheumatoid vasculitis-related neuropathy begins with the identification of key clinical criteria, including a confirmed history of RA, neurological symptoms, and prior glucocorticoid exposure. Patients meeting these conditions undergo preliminary assessment, followed by comprehensive laboratory and neuroimaging evaluations. Laboratory studies typically reveal elevated RF and ESR, decreased complement C3, and variable ANA status, while MRI in combination with MRA or CTA may demonstrate ischemic or hemorrhagic lesions accompanied by characteristic beaded stenosis or segmental occlusion. CSF analysis often shows lymphocytic pleocytosis, elevated protein, and an increased IgG index. Critical to the diagnostic pathway is the systematic exclusion of alternative etiologies such as infection, malignancy, metabolic disorders, demyelinating diseases, and other autoimmune conditions. In cases where typical radiological or laboratory features are absent yet clinical suspicion remains, a brain biopsy may be pursued for pathological confirmation. The management strategy involves initiating immunosuppressive therapy—such as rituximab or tocilizumab—with concurrent glucocorticoids and cyclophosphamide or azathioprine. Treatment response is monitored closely, and in instances of suboptimal outcome, therapy may be escalated or revised. Patients are thereafter maintained on regular follow-up, including repeat imaging and laboratory studies as indicated, to ensure sustained disease control. Future multicenter collaborations should expand datasets to refine disease characterization and develop non-invasive biomarkers.

This study has several limitations. First, the extended literature review period (1951–2025) introduces heterogeneity in diagnostic and therapeutic criteria over time, complicating direct comparisons of historical and contemporary serological data. Second, due to the patient’s critical condition upon admission, contrast-enhanced MRI was not performed for safety reasons; future studies should incorporate such imaging in eligible patients to enhance diagnostic precision. Finally, the small sample size—although inherent in rare diseases—limits statistical power and generalizability, underscoring the need for larger, multicenter collaborations to validate these findings.

## 5 Conclusions

CRV is a life-threatening RA complication requiring biopsy-confirmed diagnosis. Corticosteroid-immunosuppressant combination therapy significantly reduces mortality versus historical approaches. Key limitations include insufficient mechanistic research, the absence of non-invasive biomarkers, and the lack of evidence-based protocols. Future research should elucidate pathogenesis, develop diagnostic biomarkers, and evaluate novel biologicals.

## Glossary

RA: rheumatoid arthritis
RV: rheumatoid vasculitis
CRV: cerebral rheumatoid vasculitis
RF: rheumatoid factor
CNS: central nervous system
CEA: carcinoembryonic antigen
AFP: alpha-fetoprotein
NSE: neuron-specific enolase
PML: progressive multifocal encephalopathy
SLE: systemic lupus erythematosus
SS: Sjögren’s syndrome
ESR: erythrocyte sedimentation rate
anti-CCP: anti-cyclic citrullinated peptide
ANAs: antinuclear antibodies
AKA: anti-keratin antibody
ANCA: anti-neutrophil cytoplasmic antibody profile
anti-dsDNA: anti-double-stranded DNA antibody
LA: lupus anticoagulant
CSF: cerebrospinal fluid
EEG: electroencephalogram
MRI: magnetic resonance imaging
DWI: diffusion-weighted imaging
MRA: magnetic resonance angiography
MRV: magnetic resonance venography
SWI: susceptibility-weighted imaging
PWI: perfusion-weighted imaging
CBV: cerebral blood volume
CBF: cerebral blood flow
TTP: time to peak
Tmax: time to maximum
MRS: magnetic resonance spectroscopy
NAA: *N*-acetylaspartate
RM: rheumatoid meningitis
OR: odds ratio
