# Supplementary material for: Cerebral rheumatoid vasculitis: an integrated analysis of case presentation, literature review, and prognostic stratification
Source: Front Immunol. 2025 Oct 28;16:1683920. doi: 10.3389/fimmu.2025.1683920 (PMC12602543; doi:10.3389/fimmu.2025.1683920)
Supplement: Supplementary file 1 [file DataSheet1.docx]

Supplementary Material

# Supplementary Data

- 1. **Prognostic modeling: Receiver operating characteristic (ROC) curves evaluated RF/ESR prognostic utility (AUC >0.7 considered significant).**

We employed ROC analysis and binary logistic regression to systematically assess prognostic predictors in a cohort of 37 patients with CRV. Using a selection criterion of p < 0.10, duration of RA and ESR were identified as candidate predictors. However, both exhibited limited accuracy in discriminating outcomes (favorable versus fatal), with area under the curve (AUC) values of 0.56 for RA and 0.50 for ESR, below the predefined significance threshold of AUC > 0.70. ROC analysis of the combined factors demonstrated a marginal improvement (AUC = 0.60) but did not achieve statistically significant prognostic value (p > 0.05). These results underscore the constrained utility of single-marker approaches and emphasize the need for multicenter cohorts integrated with multivariate modeling to improve predictive performance **(Supplementary Figure 1)**.

# 1.2 References for the Literature Review

[1] PIRANI C L, BENNETT G A. Rheumatoid arthritis; a report of three cases progressing from childhood and emphasizing certain systemic manifestations [J]. Bull Hosp Joint Dis, 1951, 12(2): 335-67.

[2] SOKOLOFF L, BUNIM J J. Vascular lesions in rheumatoid arthritis [J]. J Chronic Dis, 1957, 5(6): 668-87.

[3] KEMPER J W, BAGGENSTOSS A H, SLOCUMB C H. The relationship of therapy with cortisone to the incidence of vascular lesions in rheumatoid arthritis [J]. Ann Intern Med, 1957, 46(5): 831-51.

[4] JOHNSON R L, SMYTH C J, HOLT G W, et al. Steroid therapy and vascular lesions in rheumatoid arthritis [J]. Arthritis Rheum, 1959, 2(3): 224-9.

[5] STEINER J W, GELBLOOM A J. Intracranial manifestations in two cases of systemic rheumatoid disease [J]. Arthritis Rheum, 1959, 2: 537-45.

[6] OUYANG R, MITCHELL D M, ROZDILSKY B. Central nervous system involvement in rheumatoid disease. Report of a case [J]. Neurology, 1967, 17(11): 1099-105.

[7] SIEVERS K, NISSILA M, SIEVERS U M. Cerebral vasculitis visualized by angiography in juvenile rheumatoid arthritis simulating brain tumor [J]. Acta Rheumatol Scand, 1968, 14(3): 222-32.

[8] RAMOS M, MANDYBUR T I. Cerebral vasculitis in rheumatoid arthritis [J]. Arch Neurol, 1975, 32(4): 271-5.

[9] WATSON P, FEKETE J, DECK J. Central nervous system vasculitis in rheumatoid arthritis [J]. Can J Neurol Sci, 1977, 4(4): 269-72.

[10] PACI R, GIUFFRIDA C M, MARANGOLO M, et al. Neuroradiologic picture of cerebral vasculitis in rheumatoid arthritis [J]. Neuroradiology, 1983, 25(5): 343-5.

[11] BECK D O, CORBETT J J. Seizures due to central nervous system rheumatoid meningovasculitis [J]. Neurology, 1983, 33(8): 1058-61.

[12] GOBERNADO J M, LEIVA C, RABANO J, et al. Recovery from rheumatoid cerebral vasculitis [J]. J Neurol Neurosurg Psychiatry, 1984, 47(4): 410-3.

[13] SUSCHKE H J, MEISTER P, PITZL H. [Fatal course of juvenile chronic arthritis] [J]. Klin Padiatr, 1990, 202(5): 365-7.

[14] OHNO T, MATSUDA I, FURUKAWA H, et al. Recovery from rheumatoid cerebral vasculitis by low-dose methotrexate [J]. Intern Med, 1994, 33(10): 615-20.

[15] SINGLETON J D, WEST S G, REDDY V V, et al. Cerebral vasculitis complicating rheumatoid arthritis [J]. South Med J, 1995, 88(4): 470-4.

[16] OHTA K, TANAKA M, FUNAKI M, et al. [Multiple cerebral infarction associated with cerebral vasculitis in rheumatoid arthritis] [J]. Rinsho Shinkeigaku, 1998, 38(5): 423-9.

[17] PEDERSEN R C, PERSON D A. Cerebral vasculitis in an adolescent with juvenile rheumatoid arthritis [J]. Pediatr Neurol, 1998, 19(1): 69-73.

[18] Hui, N. L., & Kang, E. Z. (2000). Rheumatoid arthritis with diffuse cerebral vasculitis: A case report [类风湿性关节炎合并弥漫性脑血管炎一例]. Journal of Beijing Military Medical, (06), 453. (In Chinese)

[19] KISS G, KELEMEN J, BELY M, et al. Clinically diagnosed fatal cerebral vasculitis in long-standing juvenile rheumatoid arthritis [J]. Virchows Arch, 2006, 448(3): 381-3.

[20] RODRIGUEZ URANGA J J, CHINCHON ESPINO D, SERRANO POZO A, et al. [Pseodotumoral central nervous system vasculitis in rheumatoid arthritis] [J]. Med Clin (Barc), 2006, 127(11): 438-9.

[21] MRABET D, MEDDEB N, AJLANI H, et al. Cerebral vasculitis in a patient with rheumatoid arthritis [J]. Joint Bone Spine, 2007, 74(2): 201-4.

[22] KURNE A, KARABUDAK R, KARADAG O, et al. An unusual central nervous system involvement in rheumatoid arthritis: combination of pachymeningitis and cerebral vasculitis [J]. Rheumatol Int, 2009, 29(11): 1349-53.

[23] CABALLOL PONS N, MONTALA N, VALVERDE J, et al. Isolated cerebral vasculitis associated with rheumatoid arthritis [J]. Joint Bone Spine, 2010, 77(4): 361-3.

[24] AKROUT R, BENDJEMAA S, FOURATI H, et al. Cerebral rheumatoid vasculitis: a case report [J]. J Med Case Rep, 2012, 6: 302.

[25] GUADALUPE LOYA-DE LA CERDA D, AVILES-SOLIS J C, DELGADO-MONTEMAYOR M J, et al. Isolated rheumatoid arthritis-associated cerebral vasculitis: a diagnostic challenge [J]. Joint Bone Spine, 2013, 80(1): 88-90.

[26] SPATH N B, AMFT N, FARQUHAR D. Cerebral vasculitis in rheumatoid arthritis [J]. QJM, 2014, 107(12): 1027-9.

[27] OZKUL A, YILMAZ A, AKYOL A, et al. Cerebral vasculitis as a major manifestation of rheumatoid arthritis [J]. Acta Clin Belg, 2015, 70(5): 359-63.

[28] RIDA M A, EL NAJJAR M, MERASHLI M. Neurologic Manifestations of Rheumatoid Arthritis: A Case of Cerebral Vasculitis Treated With Rituximab [J]. Arch Rheumatol, 2019, 34(2): 238-40.

[29] TAKEUCHI Y, MURAHASHI S, HARA Y, et al. Possible Cerebral Vasculitis in a Case with Rheumatoid Arthritis [J]. Intern Med, 2021, 60(6): 935-40.

[30] BARMAN B, SARMA K, PHUKAN P. Isolated Cerebral Vasculitis in a Patient with Rheumatoid Arthritis [J]. Neurol India, 2021, 69(4): 1097-8.

[31] EL HASBANI G, EL OUWEINI H, DABDOUB F, et al. Rheumatoid Cerebral Vasculitis in a Patient in Remission [J]. Clin Med Insights Case Rep, 2022, 15: 11795476221083114.

[32] Chen, Y., Lei, X. Y., Pu, Y., et al. (2022). A case report of clinically diagnosed rheumatoid cerebral vasculitis [临床诊断的类风湿性脑血管炎1例报告]. Journal of Apoplexy and Nervous Diseases, 39(02), 163-164. (In Chinese)

[33] FANA M, CHAO S, KATRAMADOS A M. Rituximab for prevention of strokes in cerebral rheumatoid vasculitis [J]. Clin Neurol Neurosurg, 2022, 215: 107199.

# Supplementary Figures and Tables

## Supplementary Figures


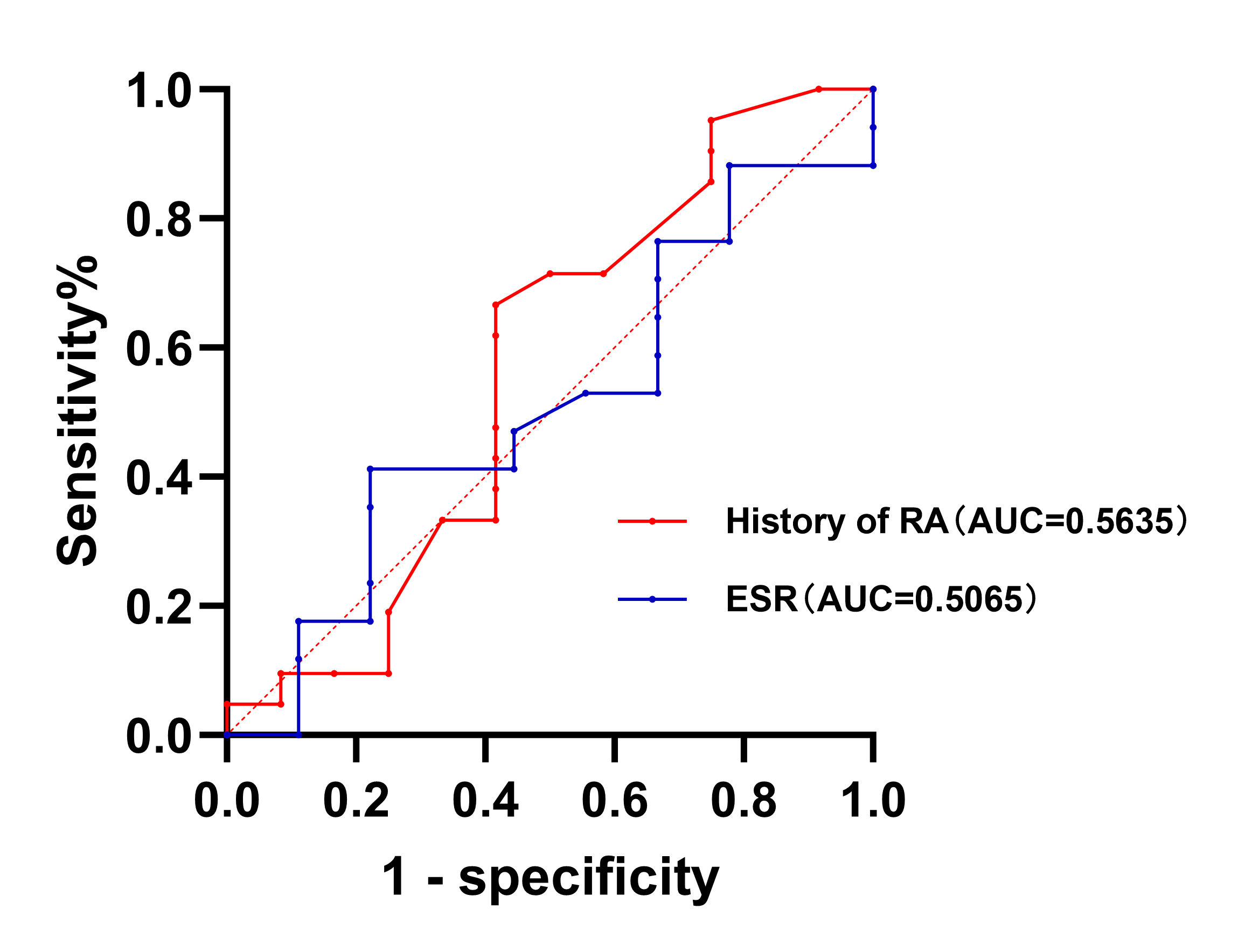


**Supplementary Figure 1.** ROC curve for the history of RA and ESR.

## Supplementary Tables

**Supplementary Table 1** Literature Review of Demographic, Clinical, Serology, Treatment, outcome of Cerebral Rheumatoid Vasculitis

| **Case** | **First author reference citation** | **Age,Sex** | **Duration**  **of RA (years)** | **RF Level** | **ESR**  **(mm/h)** | **CRP**  **(mg/dL)** | **Complement C3 (mg/dL)** | **Neurological symptom** | **Treatement** | **Outcome** |
| --- | --- | --- | --- | --- | --- | --- | --- | --- | --- | --- |
| 1 | Pirani,1951 [1] | 22,M | 16 | - | 36 | - | - | Seizure,delirium | - | Death |
| 2 | Sokoloff,1957[2] | 64,M | 30 | - | Increased | - | - | - | Steroid | Death |
| 3 | James,1957[3] | 63,F | 18 | - | 120 | - | - | Disturbance of consciousness, visual/auditory hallucinations, slurred speech, right facial weakness, left hemiplegia with right arm weakness, bilateral sensory loss | Steroid | Death |
| 4 | Johnson,1959[4] | 37,F | 1.67 | - | - | - | - | Seizure, numbness and pain in both lower limbs | Steroid | Death |
| 5 | Johnson,1959[4] | 63,M | 3 | - | Increased | - | - | Loss of consciousness, left hemiparesis, Incoherent speech, sensory impairment, memory loss | Steroid | Death |
| 6 | Steiner,1959[5] | 62,M | 20 | - | - | - | - | Fever, confusion, loss of consciousness, sluggish light reflex in the right pupil | Steroid | Death |
| 7 | Ouyang, 1967 [6] | 58,F | 30 | - | 102 | - | - | Right hemiparesis, seizure, dysarthria, loss of consciousness, diminished tendon reflexes in both lower limbs | Steroid | Death |
| 8 | Sievers, 1968[7] | 17,M | 7 | Normal | 50 | - | - | Right hemiparesis, aphasia, headache, seizure, dysgraphia | Steroid | Improvement |
| 9 | Ramos, 1975[8] | 63,M | - | - | 58 | - | - | Gerstman syndrome, blindness, loss of consciousness | - | Death |
| 10 | Watson, 1977[9] | 54,F | 20 | - | 120 | - | - | Aphasia, dysphagia, left peripheral facial paralysis, right hemiparesis, left ataxia | Steroid | Improvement |
| 11 | Paci, 1983[10] | 50,F | 6 | - | - | - | - | Loss of consciousness, left hemiparesis | Steroid | Improvement |
| 12 | Beck, 1983[11] | 52,M | 2 | 1:320 | 78 | 8.9 | Normal | Loss of consciousness, seizure, right hemihypesthesia, aphasia | Steroid, AZA | Improvement |
| 13 | Gobernado, 1984[12] | 48,F | 22 | Normal | 30 | - | 142 | Headache, left internuclear ophthalmoplegia, diplopia, bilateral pyramidal tract involvement, seizure, asymmetric weakness in all four, limbs generalized hyperreflexia, loss of consciousness | Steroid | Improvement |
| 14 | Suschke, 1990[13] | 20,M | 8 | Increased | 15 | Normal | 75 | Headache, numbness in the left limbs, dysarthria, seizure, loss of consciousness | Steroid, MTX | Death |
| 15 | Ohno, 1994[14] | 46,F | 16 | 79.9 UI/ml | 40 | 0.5 | 84 (Normal) | Dysarthria, left hemiparesis, hyperreflexia, headache | Steroid, CPA | Improvement |
| 16 | Singleton , 1995[15] | 55,F | 7 | 1:10240 | 58 | 0.4 (Normal) | 116 | Mental status changes, dysarthria, ataxia, seizure, urinary incontinence, left hemiparesis | Steroid, CPA, AZA | Death |
| 17 | Ohta, 1998[16] | 64,F | 7 | 415UI/ml | 116 | - | - | Delirium, aphasia, weakness in all four limbs, higher cortical dysfunction | Steroid | Improvement |
| 18 | Pedersen, 1998[17] | 16,F | 6 | Normal | 124 | - | 253 | Right hemiparesis, right central facial palsy and tongue deviation | Steroid, MTX | Improvement |
| 19 | Hui, 2000[18] | 45,F | 1 | 1:640 | 86 | - | - | Headache, vomiting, seizure, dysarthria | Steroid | Improvement |
| 20 | Kiss, 2006[19] | 51,F | 39 | - | 70 | 56 | - | Left hemiparesis, loss of consciousness | Steroid, IVIg | Death |
| 21 | Rodriguez, 2006[20] | 49,F | 10 | 127.4UI/ml | 50 | - | - | Aphasia, hemianopia | Steroid, CPA | Improvement |
| 22 | Rodriguez, 2006[20] | 70,F | - | 57.6UI/ml | - | - | - | Seizure | Steroid, CPA | Improvement |
| 23 | Mrabet, 2007[21] | 59,F | 20 | - | 135 | 5.7 | - | Headache, diplopia, ataxia | Steroid, CPA, MTX | Improvement |
| 24 | Kurne, 2009[22] | 63,F | 12 | - | - | - | - | Headache, quadriplegia, dysarthria, cognitive impairment, seizure, loss of consciousness | Steroid, CPA, Immunosuppressants | Improvement |
| 25 | Caballol, 2010[23] | 71,F | 15 | 1:160 | 79 | 24 | - | Headache, dysarthria, left hemiparesis | Steroid | Improvement |
| 26 | Akrout, 2012[24] | 52,F | 9 | 512UI/ml | 27 | 1.4 | Normal | Headache | Steroid, MTX | Improvement |
| 27 | Guadalupe, 2013[25] | 47,F | 11 | 1:3200 | 70 | 5.9 (**Reference Range** 0-5) | - | Headache, Seizure, loss of consciousness | Steroid, CPA, IVIg | Improvement |
| 28 | Spath, 2014[26] | 52,F | 20 | - | - | - | Normal | Headache, hemianopia, ataxia, aphasia, left hemiparesis, loss of consciousness | Steroid, CPA, AZA | Improvement |
| 29 | Ozkul, 2015[27] | 30,F | 20 | 42.9UI/ml | 64 | 28.5 (**Reference Range** 0-6) | - | Headache, weakness in the left face and upper limb | Steroid, AZA | Improvement |
| 30 | Ozkul, 2015[27] | 52,M | 29 | 82.9UI/ml | 116 | 32 | - | Dysarthria, mild weakness in the right upper limb | Steroid, CPA | Improvement |
| 31 | Rida, 2019[28] | 61,F | - | - | - | - | - | - | Steroid, RTX | Improvement |
| 32 | Takeuchi, 2021[29] | 81,F | 4 | 829UI/ml | 73 | 0.99 | 61 | Fever, loss of consciousness, Kernig's sign, right hemiparesis | Steroid, CPA | Death |
| 33 | Barman, 2021[30] | 37,F | 12 | - | 77 | 1.6 | - | Headache, sleep disturbance, mild tremor, Seizure | Steroid, MTX | Improvement |
| 34 | El Hasbani, 2022[31] | 59,F | 7 | Normal | 27 | 0.09 | Normal | Headache, ataxia, recent memory impairment | Steroid, RTX | Improvement |
| 35 | Chen Yi , 2022[32] | 54,M | - | Normal | 75 | 8.54 | - | Headache, psychobehavioral abnormalities, seizure, right hemiparesis | Steroid, CPA | Improvement |
| 36 | Fana, 2022[33] | 44,F | 14 | Increased | - | - | Normal | Sensorineural hearing loss in the right ear, ataxia, sensory impairment in the left limbs | Steroid, RTX | Improvement |
| 37 | Present case | 64,F | 30 | 563UI/ml | 56 | 4.43 | 81 | Loss of consciousness, left hemiparesis, aphasia, seizure | Steroid, CPA | Improvement |

RA: rheumatoid arthritis, RF: rheumatoid factor, ESR: erythrocyte sedimentation rate, CRP: C - reactive protein, MTX: methotrexate, CPA: y-lophosphamide, IVIg: intravenous immunoglobulin, AZA: azathioprine, RTX: rituximab

**Supplementary Table 2** Literature Review of Abnormal lesions in CT or MRI, Cerebrovascular findings, Pathological findings of brain of Cerebral Rheumatoid Vasculitis

| **Case** | **Abnormal lesions in CT or MRI** | **Cerebrovascular findings** | **Pathological findings of brain** |
| --- | --- | --- | --- |
| 1 | - | - | Widespread polyarteritis nodosa (involving the basilar artery); softening of adjacent brain tissue |
| 2 | - | - | Widespread nodular necrotizing peripulmonary inflammation |
| 3 | - | Basilar necrotizing arteritis, with symmetrical spindle thickening of the vessel wall | Widespread nodular polyarteritis; thrombosis of the basilar artery with right pontine infarction, stenosis of cerebral vascular wall |
| 4 | - | Cerebral artery necrotizing arteritis | Diffuse necrotizing polyarteritis; nodular polyarteritis type |
| 5 | - | - | Cerebral vasculitis; extensive systemic arteritis. Manifested as extensive lymphocyte infiltration and perivascular fibrosis |
| 6 | - | Secondary vasculitis with ischemia of cerebral cortex and white matter | Vasculitis with secondary ischemic changes in cerebral cortex and white matter; Beads on a string-like change in basal vessels; "Fibrinoid" and "necrotic" rheumatoid nodules scattered throughout the dura mater |
| 7 | - | - | Cerebral vasculitis |
| 8 | - | Vascular caliber changes | - |
| 9 | - | - | Cerebral vasculitis, similar to polyarteritis nodosa; focal granular necrosis of cerebral cortex, focal severe chronic vasculitis with fibrinoid necrosis in cerebral cortex |
| 10 | - | - | Cerebral necrotizing arteritis; bilateral frontal lobe and right pontine hematoma |
| 11 | Multiple ischemic areas in left temporal lobe and left and right frontal and parietal lobes | Narrowing and dilation of branches of the anterior cerebral artery, medial and posterior internal frontal arteries and the ascending frontal artery; alteration in diameter of the medial internal frontal branch of the right anterior cerebral artery  Cervical right carotid angiography showed changes in the diameter of the medial internal frontal branch of the anterior cerebral artery | - |
| 12 | Diffuse enhancement of bilateral anterior frontal cortex | - | Cerebral vasculitis |
| 13 | Small hemorrhage in right temporal lobe, cerebral white matter lesion | Bilateral carotid and vertebral artery segmental stenosis. The main affected sites are middle cerebral artery, posterior cerebral artery and posterior inferior cerebellar artery | - |
| 14 | - | - | Cerebral cerebral vasculitis |
| 15 | Basilar artery stenosis, right pontine ischemic changes | Multiple discrete segmental stenosis and dilation of the vertebrobasilar artery, with more severe involvement on the right side | - |
| 16 | Right pontine infarction (significant multifocal abnormalities in cerebral white matter) | normal | Inflammatory infiltration and fibrosis were observed perivascularly in the periventricular white matter, pons and right hippocampus. The arteries at the base of the brain showed moderate atherosclerosis. |
| 17 | New infarction foci occurred in the temporal and parietal lobes of both sides | normal | - |
| 18 | - | Vasculitis of the anterior and middle cerebral arteries (the beads and narrowing of the Willis ring vessels, involving the anterior and middle cerebral arteries) | - |
| 19 | Left basal ganglia cerebral ischemic change | - | - |
| 20 | - | - | Cyanotic necrosis of small and microarteries in cerebral white matter, perivascular fibrosis and lymphocyte infiltration |
| 21 | Left frontal lobe white matter lesion | normal | - |
| 22 | Left occipital cortex lesion | - | Non-granulomatous lymphocytic vasculitis |
| 23 | There are punctate hypersignal areas in the periventricular cortex bilaterally, indicating cerebral hemispheric vasculitis | Long segment stenosis of right internal carotid artery and beaded stenosis of left internal carotid artery | giant cell arteritis |
| 24 | The abnormal enhancement of the leptomeninges and the high signal of the corticospinal subarachnoid space with FLAIR are consistent with the manifestations of dural meningitis.  Chronic hemorrhagic right cortical infarction is accompanied by continuous diffuse white high signal lesions | The mid-segment of the right anterior cerebral artery and the distal segment of the left anterior cerebral artery showed reduced diameter and irregular contour. Cerebral angiography revealed irregular, long-segment and multifocal stenosis of the vessel walls, indicating extensive vasculitis involving the right middle artery and two anterior arteries. | - |
| 25 | There was a wide range of high signal shadows in the right frontal, parietal and temporal white matter, and the right meninges showed increased signal | normal | There was necrotizing and lymphocytic vasculitis in both the brain membrane and brain parenchyma, with lymphocyte infiltration involving all layers of the vascular wall and signs of vascular occlusion |
| 26 | T2WI high intensity signal at the junction of frontal and parietal cortex and subcortical cortex | Bilateral vasculitis | - |
| 27 | High signal white matter lesions in the frontal and parietal lobes, hippocampus and cerebellum | nodular vasculitis | - |
| 28 | Multiple large areas of new non-hemorrhagic infarction were found in the right occipital, right frontal lobe, left occipital lobe and left hemispheric center | Bilateral extensive proximal cerebral artery stenosis with bead-like and stenotic distal anterior cerebral arteries | Small vessels with inflammatory infiltration of the vascular wall. Lymphocyte infiltration and focal destruction of the vascular wall |
| 29 | The frontal lobe region showed hyperintensity due to acute ischemia, with hyperintensity also observed in the left temporal region and right frontotemporal and occipital regions. Three days after admission, lacunar acute ischemic lesions were found in the right central prefrontal gyrus, bilateral frontal and parietal lobes, and the corpus callosum region; subarachnoid hemorrhage | Cerebrovascular inflammation (right middle cerebral artery with reduced diameter and irregular contour) | - |
| 30 | Acute ischemic changes in bilateral occipital lobes | Cerebral vasculitis (reduced diameter and irregular contour of the right MCA) | - |
| 31 | There are multiple converging high fluid attenuation reversal recovery (FLAIR) signals in the periventricular white matter, hippocampal loops and bilateral cerebral hemisphere regions | - | - |
| 32 | Multiple cerebellar ischemias; left subcortical lesions in the frontal lobe and basal ganglia, left medial temporal lobe edema, left striatal artery enhancement. New lesion in the left medial frontal lobe. | No cerebral vascular stenosis or occlusion was observed. The left middle cerebral artery perforating branch was enhanced | - |
| 33 | High intensity lesions in the deep and subcortical white matter of the right occipital lobe | The M1 segment of the left middle cerebral artery showed irregular beaded stenosis | - |
| 34 | Ischemic changes in bilateral temporal and insular lobes | Lack of distal branch of left middle cerebral artery and mild irregularity of M1 segment of left middle cerebral artery | - |
| 35 | Infarction foci in the left occipital-occipital junction, abnormal signal shadows in the left occipital-cortical area | normal | - |
| 36 | There was old cerebral infarction in the middle cerebellum on the right side, acute infarction in the left cerebellar foot, left basal ganglia and left temporal lobe. Acute infarction of the left posterior lateral medulla oblongata. Chronic infarction of the right pons. High signal and nonspecific lesions in the right parietal lobe and left frontal lobe suggest subacute infarction. | Bilateral posterior cerebral artery and proximal branch of left middle cerebral artery M2 stenosis, bilateral superficial temporal artery wall linear enhancement. Left vertebral artery hypoplasia. | - |
| 37 | Multiple cerebral infarction foci were observed in the paraventricular and basal ganglia regions. Large patchy abnormal signal lesions were identified in the right parietal, temporal, and occipital lobes along with the basal ganglia, while a patchy hemorrhagic lesion was detected in the left occipital lobe. Additionally, subarachnoid hemorrhage was confirmed in the right parietal lobe. | Intracranial vascular atherosclerotic changes, segmental stenosis of the M1 segment of the left middle cerebral artery, and beaded stenosis of the bilateral anterior cerebral arteries | Gliosis was observed in the brain parenchyma with focal softening foci; partial necrosis of cerebral vascular walls and infiltration of lymphocytes, plasma cells and a small number of neutrophils were observed |

**Supplementary Table 3** Baseline Characteristics and Clinical Outcomes in Patients with CRV（n=37）

| **Variable** | **Statistical value** | **Remark** |
| --- | --- | --- |
| **Demography** | | |
| Gender (Female/Male) | 27 (73.0%) / 10 (27.0%) | The proportion of women is significantly higher than that of men（χ²=7.81, p<0.01） |
| Age (years) | 51.2±15.2（range：16-81） | The median age was 51 years (IQR: 46-63).  Female 53 years (IQR: 46.5–62)  Male 47 years (IQR: 29.5–62.8)  There was no significant difference in the age distribution between men and women, p>0.05 |
| RA-related characteristics | | |
| Duration of RA (years) (n=33). | 14.8±9.8 (range: 1-39). | The duration of the disease ≥ 10 years accounted for 60.7% (20/33)  Median disease duration of 12 years (IQR: 7-20) |
| RF positivity rate (n=21) | 16/21 (76.2%) | —— |
| RF level (n=9) | 302.2±256.2UI/ml（range：42.9–829UI/ml） | Median RF levels127.4UI/ml （IQR：63.28–487.75） |
| **Laboratory indicators** | | |
| ESR is elevated（n=27） | 29/29 (100%) (mean: 67.78±53.42 mm/h). | —— |
| Decreased complement C3 (n=12) | 3/12 (25%) (mean: 116±56.72 mg/dL). | —— |
| **Clinical presentation** | | |
| Neurological symptoms（n=35） | Altered mental status (54.3%), paralysis (45.7%), epilepsy (42.9%), headache (42.9%), seizures (34.3%), cranial nerve palsy (25.7%), gait disturbance (22.9%), aphasia (22.9%), dementia (14.3%), blindness (8.6%) | It usually presents with multifocal neurological deficits |
| Skin nodules | 6/37 (16.2%) | Suggests systemic vasculitis |
| **Imaging features** | | |
| Ischemic lesions（n=24） | 14/24 (58.3%) | The frontotemporal occipital lobe (62.3%) and basal ganglia (28.6%) were the main ones  Multifocal distribution (71.4%), bilateral asymmetry (64.3%) |
| Hemorrhagic lesions（n=24） | 4/24 (11.4%) | Subarachnoid hemorrhage (75%), cerebral parenchymal hemorrhage (50%) |
| Abnormal white matter signals（n=24） | 8/24 (33.3%) | FLAIR hyperintensity was predominant, mostly involving frontoprietal white matter (62.5%) |
| Cerebrovascular abnormalities（n=25） | 19/25 (76.0%) | The main manifestations are beaded stenosis and segmental occlusion  The middle cerebral artery (47.4%) and the anterior cerebral artery (15.8%) were the most commonly affected |
| Other features:（n=24） | 5/24 (20.8%) | It needs to be differentiated from tumor and infection |
| **Pathologic features (n=18).** | | |
| Necrotizing vasculitis | 9/18 (50.0%) | The remainder present with non-necrotizing lymphocytic infiltrates |
| Lymphocytic/plasmacytic infiltration | 17/18 (94.4%) | Mixed infiltrates (lymphocyte-predominant) indicate chronic immune activation |
| Neutrophil infiltration | 2/18 (11.1%) |  |
| Fibrinoid necrosis | 6/18 (33.3%) | —— |
| Fibrosis and amyloidosis | 4/18 (22.2%) | —— |
| Immune complex deposition (n=3) | 3/3 (100%) | Positive for IgG and complement C3 |
| **Treatment and outcomes** | | |
| glucocorticoids immunosuppressants (n=36). | 22/35 (62.9%) | Cyclophosphamide (54.5%), methotrexate (22.7%), azathioprine (18.1%), rituximab (9.1%), immunoglobulin (9.1%) |
| Clinical outcomes | Improved: 25/37 (67.7%); Deaths: 12/37 (32.4%) | Eight of the deaths were not treated with immunosuppressants |
